# Perioperative diltiazem or nitroglycerin in on-pump coronary artery bypass: A systematic review and network meta-analysis

**DOI:** 10.1371/journal.pone.0203315

**Published:** 2018-08-30

**Authors:** Yirui Hu, Xinbei Yang, Li Zhang, Xianren Wu, Anastasia Yian Liu, Joseph A. Boscarino, H. Lester Kirchner, Alfred S. Casale, Xiaopeng Zhang

**Affiliations:** 1 Biomedical & Translational Informatics, Geisinger Medical Center, Danville, Pennsylvania, United States of America; 2 Division of Anesthesiology, Geisinger Medical Center, Danville, Pennsylvania, United States of America; 3 Department of Cell and Systems Biology, University of Toronto, Toronto, Canada; 4 Department of Epidemiology and Health Services Research, Geisinger Medical Center, Danville, Pennsylvania, United States of America; 5 Geisinger Heart Institute, Geisinger Wyoming Valley Medical Center, Wilkes Barre, Pennsylvania, United States of America; Bern University Hospital, SWITZERLAND

## Abstract

**Background:**

Arterial graft spasm is a severe complication after coronary artery bypass graft (CABG). Among numerous potential antispasmodic agents, systemic application of diltiazem and nitroglycerin had been investigated most frequently over the past three decades. However, it remains inconclusive if either or both agents could improve patient outcomes by preventing graft spasm when applied perioperatively, and, if so, which one would be a better choice. The current systematic review and network meta-analysis aims to summarize the data from all available randomized clinical trials of perioperative continuous intravenous infusion of diltiazem and/or nitroglycerin in patients undergoing on-pump CABG in order to define and compare their roles in graft spasm prevention and their impacts on perioperative outcomes.

**Methods:**

We searched Ovid Medline, PubMed, CINAHL, Google Scholar and Cochrane Center for randomized controlled trials that reported outcome effects of perioperative continuous intravenous infusion of diltiazem and/or nitroglycerin in patients undergoing elective on-pump CABG. Conventional meta-analyses were conducted to evaluate the pairwise comparisons (diltiazem vs. placebo; nitroglycerin vs. placebo; diltiazem vs. nitroglycerin) on perioperative outcomes. Network meta-analyses were implemented to compare the three regimens through direct and indirect comparison.

**Results:**

Twenty-seven studies involving 1,660 patients were included. Pairwise and network meta-analyses found no significant difference in mortality among the groups. There are four studies that reported blood flow measurements of internal mammary artery grafts intraoperatively after dissecting or immediately after distal anastomosis while patients were on continuous intravenous infusion of diltiazem and nitroglycerin. Although insufficient for data synthesis, the measured results from all four studies suggest that both diltiazem and nitroglycerin significantly increased blood flow of arterial grafts compared to placebo. For other perioperative outcomes, compared to diltiazem, patients that received nitroglycerin had higher odds of postoperative atrial fibrillation (OR = 2.67, 95% CI: 1.15 to 6.24) and higher peak serum cardiac enzymes. Patients that received placebo had higher odds of atrial fibrillation (OR = 3.00, 95% CI: 1.18 to 7.63) and lower odds of requiring inotrope support (OR = 0.19, 95% CI: 0.04 to 0.73) compared to diltiazem. Data from the network meta-analysis indicated that diltiazem had significantly lower odds of postoperative atrial fibrillation compared to nitroglycerin (OR = 0.39, 95% CI: 0.18 to 0.85). In fact, the rank from highest to lowest rates of postoperative atrial fibrillation was placebo>nitroglycerin>diltiazem. The rank from highest to lowest odds of requiring inotropic support is nitroglycerin> diltiazem>placebo. However, placebo had significantly higher odds of postoperative myocardial infarction than diltiazem (OR = 4.51, 95% CI: 1.34 to 15.25). The rank from highest to lowest odds of postoperative myocardial infarction, transient cardiac ischemic event and atrial fibrillation is placebo>nitroglycerin>diltiazem.

**Conclusion:**

Compared to nitroglycerin and placebo, perioperative continuous intravenous infusion of diltiazem had stronger protective effects against postoperative ischemic cardiac injuries and atrial fibrillation although patients may need more inotropic support. The increased blood flow from diltiazem use in arterial grafts may potentially contribute to the drug’s outcome benefits.

## Introduction

Coronary artery bypass graft (CABG) has been established as the standard procedure of revascularization for patients with multi-vessel coronary artery disease (CAD). The application of autologous grafts on arteries including internal mammary arteries (IMA), radial arteries (RA), gastroepiploic arteries and inferior epigastric arteries has greatly improved the short- and long-term outcomes of CABG. Compared with saphenous vein grafts (SVGs), arterial grafts have significantly higher graft patency over time. In fact, some authors proposed using total arterial grafts to replace SVGs, although, currently, there is insufficient clinical follow-up data to support this strategy[1–3].

One of the challenging issues with arterial grafts is graft spasm leading to intra- and/or post-operative myocardial ischemia and cardiac arrhythmia. While arterial graft spasm was first reported clinically in 1987[4,5], its mechanisms remain unclear. However, studies have indicated that it is likely multifactorial, including mechanical stimulation from graft manipulation, vasoactive molecules released from activated endothelial cells during cardiac reperfusion, the application of certain vasoactive drugs and so on. These insults cause increased extracellular influx and intracellular sarcoplasmic release of calcium ions through different signaling pathways. A previous meta-analysis indicated that perioperative application of different calcium channel blockers (CCBs) had significant outcome benefits for patients undergoing all types of cardiac surgeries requiring cardiopulmonary bypass (CPB)[5]. Topical application of CCBs on isolated IMAs and RAs consistently showed evidence of vasodilatation and increased blood flow[6,7].

As our understanding of its mechanisms improves, increasing effort has been devoted to developing effective pharmacological interventions for preventing graft spasm after CABG. The most investigated agents for this purpose are CCBs and nitrates. There are also clinical studies for alpha-1 blockers[8], phosphodiesterase III inhibitors, e.g. milrinone[9,10], calcium sensitizers, e.g. levosimendan [11], potassium channel openers, e.g. aprikalim[12], and prostacyclin analogues, e.g. iloprost[13]. These investigational drugs were applied in various ways: topically or intra-graft injection[6,14], systemically through bolus and/or intravenous (IV) infusion, or mixed in cardioplegia during CPB[15,16].

Diltiazem (DILT), a benzothiazepine-type CCB, is well known for relieving coronary spasm while uniquely being able to promote vasodilation without rebound tachycardia. Nitroglycerin (NTG) infusion is also one of the first line therapeutic interventions of unstable angina; injection of NTG into IMA grafts showed potent vasodilatation[17]. This systematic review and network meta-analysis therefore aims to summarize the available outcome data from clinical trials involving perioperative continuous IV infusion of DILT and/or NTG in patients undergoing on-pump CABG, and compare the drugs’ effects on graft blood flow, perioperative mortality, perioperative hemodynamic stability, ventricular functions, postoperative myocardial infarction (MI), new onset cardiac arrhythmias and requirement of inotropic support.

## Materials and methods

### Literature search strategy

**Fig 1:**
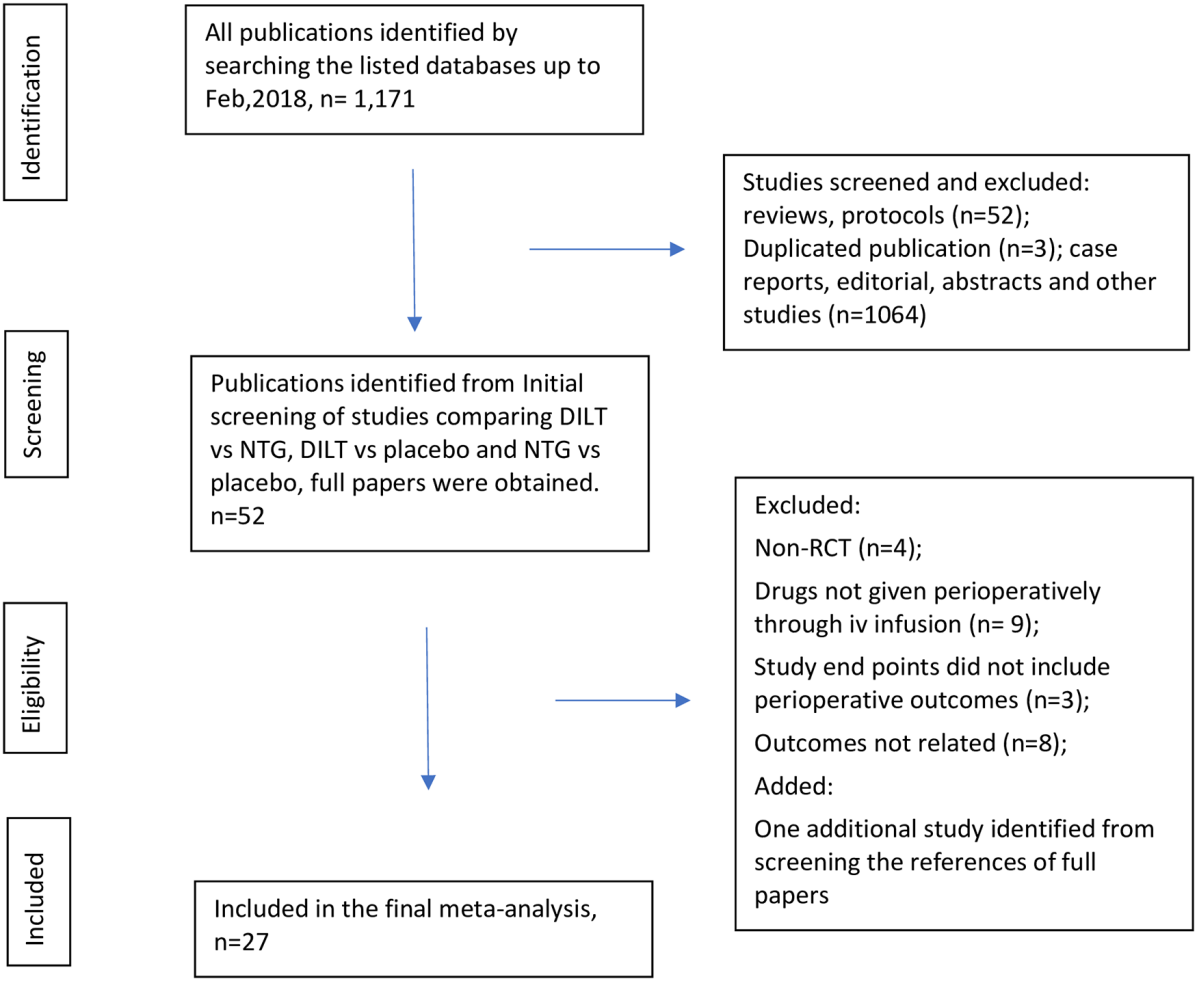
PRISMA flowchart. The literature search and study enrollment. Flow chart for literature enrollment from identification to final synthesis according to the PRISMA protocol. DILT = diltiazem; NTG = nitroglycerin; RCT = randomized controlled trial.

This systematic review and network meta-analysis was conducted based on the criteria of the Preferred Reporting Items for Systematic reviews and Meta-analysis (PRISMA) statement[18]. As shown in Fig 1, a comprehensive literature search was conducted using databases Ovid Medline, PubMed, EMBASE, CINAHL, Google Scholar and Cochrane Central Registry of Controlled Trials. These databases hosted papers published between 1946 and the end of February 2018. The search terms used were: “cardiac surgery”, “thoracic surgery”, “cardiac surgical procedures”, “coronary artery bypass”, “cardiopulmonary bypass”, “calcium channel blocker”, “diltiazem”, “nitrates”, “nitroglycerin”. The detailed search criteria applied in Ovid Medline are shown in S1 Table. The included studies should be randomized controlled trials (RCTs) or prospective cohort studies investigating effects of perioperative continuous IV infusion of DILT and/or NTG on arterial graft flow and perioperative outcomes in adult patients undergoing on-pump CABG. The seven reasons for a study to be excluded from final enrollment are: (1) the study is retrospective, (2) the study’s subjects were not limited to adult patients, (3) the studied drugs were administered topically, through intra-graft injection or mixed in cardioplegia, (4) the study was of non-cardiac surgeries, off-pump CABG or cardiac surgical procedures necessitating cardiac chamber opening, (5) the study was published as an abstract, a case report, case series, letter to the editor, editorial, narrative or systematic review, meta-analysis, or a study that did not report the investigated outcomes (6) the paper is a duplicated publication of an enrolled study, (7) the study was not approved by an institutional review board.

The initially-identified studies were screened by one reviewer (XZ) for RCTs or prospective cohort studies. The screened publications were verified by the second reviewer (XY) before being enrolled into the final systematic review and network meta-analysis. We also manually searched through the references of the enrolled papers for potential studies not captured by the database searching strategy.

### Data extraction

Data from enrolled studies were extracted into a spreadsheet by two reviewers (XZ, XY), independently. This data included sample size, geographic regions, age, sex, race/ethnicity and outcomes regarding cardiac functions. Disputations during the process of literature searching and data extraction were resolved upon reaching consensus through discussions with all the co-authors.

The complete texts of the enrolled studies were inspected by the authors independently and the following outcome parameters were extracted: patient characteristics, measurement of arterial graft blood flow, perioperative mortality, incidence of post-operative MI (post-MI), postoperative atrial fibrillation (A-fib), transient cardiac ischemic event (TIE), inotrope requirement, peak postoperative cardiac enzymes and hemodynamic parameters, such as heart rate (HR), cardiac index (CCI), mean blood pressure (mBP) and mean pulmonary arterial pressure (mPAP) and pulmonary artery wedge pressure (PAWP).

The primary outcomes are graft blood flow alteration and perioperative mortality. The remaining perioperative outcomes were further categorized into: (1) cardiac protection outcomes including post-MI, TIE, A-fib and postoperative peak cardiac enzymes. (2) cardiac function outcomes including HR, mBP, mPAP, PAWP and requirement of inotropic support.

Although the results from the individual studies were reported in different formats, the continuous variables were all converted in the data extraction spreadsheet prepared for future meta-analysis, if necessary. The values were converted to mean and standard deviation, the dichotomous variables for frequency of events.

### Quality assessment

**Fig 2:**
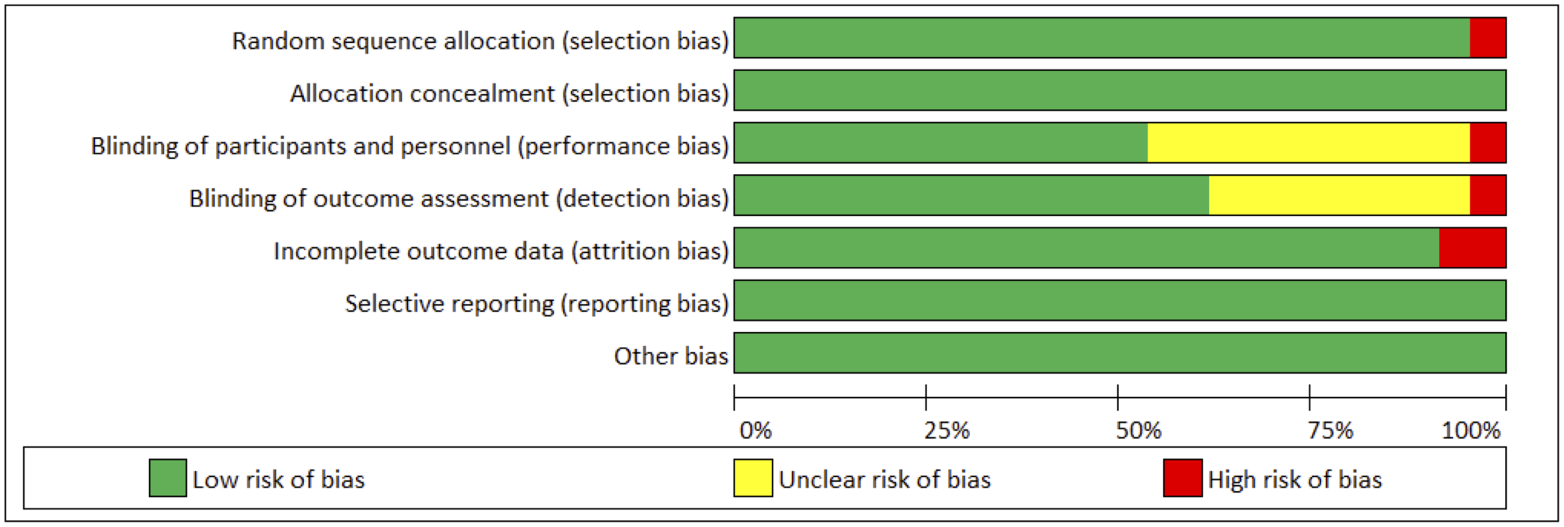
Quality assessment of enrolled clinical trials. Quality assessment was conducted using the Cochrane risk of bias assessment tool. Risk of bias assessment for included studies in meta-analysis was classified as “high”, “low” or “unclear”.

The RCTs were evaluated by two reviewers using the Cochrane risk of bias assessment tool[19], which evaluated 6 domains including random assignment, allocation concealment, blinding of participants, incomplete outcome data, selective outcome reporting and other sources of bias. The assessment of “yes,” “no,” or “unclear” was assigned to each domain for respective designation of a low, high, or unclear risk of bias. If “unclear” or “no” was assigned to one or less domains, the study was evaluated as having a low risk of bias. If over four domains were assigned “unclear” or “no”, the study was evaluated as having a moderate risk[20], see Fig 2.

### Statistical analyses

First, the pairwise meta-analyses were conducted for each included outcome using random-effects model. Odds ratios (ORs) and 95% confidence intervals (95% CIs) were estimated for binary outcomes. Mean differences (MDs) and 95% CIs were calculated for continuous outcomes. The pooled OR is considered statistically significant if 95% CI did not contain 1, and the pooled MD is considered statistically significant if 95% CI did not contain 0. Individual and pooled estimates were illustrated using forest plots.

Second, network meta-analysis (NMA) was performed to incorporate multiple comparisons for each available outcome using multivariate meta-analyses, where the within-network heterogeneity was assumed common and the heterogeneity variance was estimated using restricted maximum likelihood (REML). For all three pairwise comparisons (closed triangle loop), both direct and indirect comparisons were integrated to evaluate the effect sizes (ORs, MDs) and 95% CIs. For those outcomes with any two pairwise comparisons available (open triangle loop), indirect estimates for the third pairwise comparison were estimated (ORs, MDs and 95% CIs).

Global test for inconsistency was performed using the Wald test statistic, which follows a chi-squared distribution under the consistency assumption[21]. P-value greater than 0.05 indicates no evidence of inconsistency. The rank probability of three treatment effects were computed using the surface under the cumulative ranking curve (SUCRA)[22]. Publication bias was evaluated using funnel plots. Sensitivity analysis was conducted by excluding studies with extreme results, defined as larger than twice or smaller than half of the pooled results. All analyses were conducted using Stata 14 (Stata Corp, College Station, TX).

## Results

### Baseline characteristics for included studies

A total of 1,660 patients were recorded in the 27 included studies. The clinical characteristics are shown in Table 1. Twenty-three trials belonged to two-arm trials, three were in the category of three-arm trials and one was in the category of four-arm trials.

**Table 1:** Study characters of enrolled clinical trials.

Authors, years, journals		Sample size				Arterial Grafts		Drug application			Drug dosage	
NMA study lists	Journal	Total	DILT	NTG	Placebo	IMA	Radial	Drug starting	Drug ending	Drug route	DILT dosage	NTG dosage
Donegani 1986[23]	Thorac cardiovas Surgeon	40	20	20		not specified		induction GA	48 h after releasing aortic cross clamp	IV infusion	0.5~3.0 mcg/kg/min	0.5 ~1.5 mcg/kg/min
Hannes 1993[24]	Eur J Cardiothorac Surg	91	44	47		Yes		initiation of CPB	24 h after releasing aortic cross clamp	IV infusion	0.1 mg/kg/h	1 mcg/kg/min
Seitelberger 1994[25]	J Thorac Cardiovasc Surg	120	60	60		Yes		initiation of CPB	24h after releasing of aortic cross clamp	IV infusion	0.1 mg/kg/h	1 mcg/kg/min
Hannes 1995[26]	European Heart Journal	66	31	33		Yes		initiation of CPB	24 h after releasing aortic cross clamp	IV infusion	0.1 mg/kg/h	1 mcg/kg/min
Keilich 1997[27]	International Journal of Angiology	211	104	107		Yes		initiating CPB	24 h after releasing aortic cross clamp	IV infusion	0.1 mg/kg/h	1 mcg/kg/min
Malhotra 1997[28]	Eur J Cardiothorac Surg	71	34	37		Yes		initiation of CPB	24 h after starting drug infusion	IV infusion	0.1 mg/kg/h	1 mcg/kg/min
Lischke 1997[29]	Anesthetist	55	29	26		not specified		before induction of GA	reach ICU postoperatively	IV bolus and infusion	0.15 mg/kg, then 3mcg/kg/min	1 mcg/kg/min
Shapira 2000[30]	Ann Thorac Surg	161	77	84		Yes		induction GA	24h post operatively	IV infusion, oral	0.1mg/kg/min	0.1 mcg/kg/min
Lassnigg 2001[31]	Wien Klin Wochenschr	49	24	25		Yes		initiation of CPB	24 h post op	IV infusion	0.1 mg/kg/h	1 mcg/kg/min
Hirnle 2000[32]	kardiol Pol	49	24	25		Yea		48 h before CABG	24 h post op	oral and IV infusion	0.1mg/kg/min	1mg/h
Zhang 2003[33]	Natl Med J China	40	20	20		Yes		initiation of CPB	24 h after releasing of aortic cross clamp	IV infusion	0.1 mg/kg/h	1mcg/kg/min
Tabel 2004[34]	Eur J Cardiothorac Surg	60	30	30		Yes		Sternotomy	after second flow measurement	IV infusion	0.05~0.1 mg/kg/h	0.25~2.5 mcg/kg/min
Colson 1992[35]	J Cardiothorac Vasc Anesth	29	15		14	not specified		induction of GA		IV infusion	2ug/kg/min	
Zanardo 1993[36]	J Cardiothorac Vasc Anesth	24	12		12	not specified		induction of GA	24 h post op	IV infusion	2mcg/kg/min	
Amano 1995[37]	Chest	23	13		10	not specified		Sternotomy	not specified	IV bolus, infusion and oral	0.1 mg/kg bolus, 2 mcg/kg/min until unclamp, then oral 30mg q8h	
Babin-Ebell 1996[38]	Eur J Cardio-thoracic Surg	70	33		37	Yes		induction GA	72h after releasing aortic cross clamp	IV infusion	0.1 mg/kg/h	
Yavuz 2002[39]	Med Sci Monit	30	15		15	Yes		24 h pre-op	48 h post op	IV infusion	2 mcg kg/min	
Fansa 2003[40]	Med Sci Monit	30	15		15	Yes		initiation of CPB	conclusion of CPB	IV infusion		
Erdem 2015[41]	Bra J Cardiovas Surg	140	70		70	Yes	yes	induction of GA		IV infusion	2.5 mcg/kg/min	
Thomson 1984[42]	Anesthesiology	20		9	11	not specified		before induction of GA	until opening of pericardium	IV infusion		0.5 mcg/kg/min
Gallagher 1986[43]	Anesthesiology	81		41	40	not specified		initiating CPB	no specified	IV infusion		1 mcg/kg/min
Withington 1988[44]	European Heart Journal	14		7	7	not specified		releasing cross clamp	not specified	IV infusion		1 mcg/kg/min
Lell et al. 1993[45]	J Card Surg	30		20	10	Yes		Induction of GA	initiation of CPB	IV infusion		1 or 2 mcg/kg/min
Knothe 1993[46]	Herz	30		15	15	Yes		induction of GA	after releasing aortic cross clamp	IV infusion		1.5 mcg/kg/min
Apostolidou 1999[47]	Ann Thorac Surg	47		30	17	Yes		after releasing aortic cross clamp	24 h post op	IV infusion		0.5~1 mcg/kg/min
Chen 2000[48]	Chinese Journal of Surgery	40		20	20	not specified		30 mins before induction of GA	24 h post op	IV infusion		10ug/kg/min
Zvara 2000[49]	J Cardiothorac Vasc Anesth	39		20	20	Yes		induction of GA	6 h after extubation	IV infusion		2 mcg/kg/min

NMA, network meta-analysis; CABG, coronary artery bypass graft; CPB, cardiopulmonary bypass; GA, general anesthesia; IV, intravenous.

### Quality of enrolled studies

**Fig 3:**
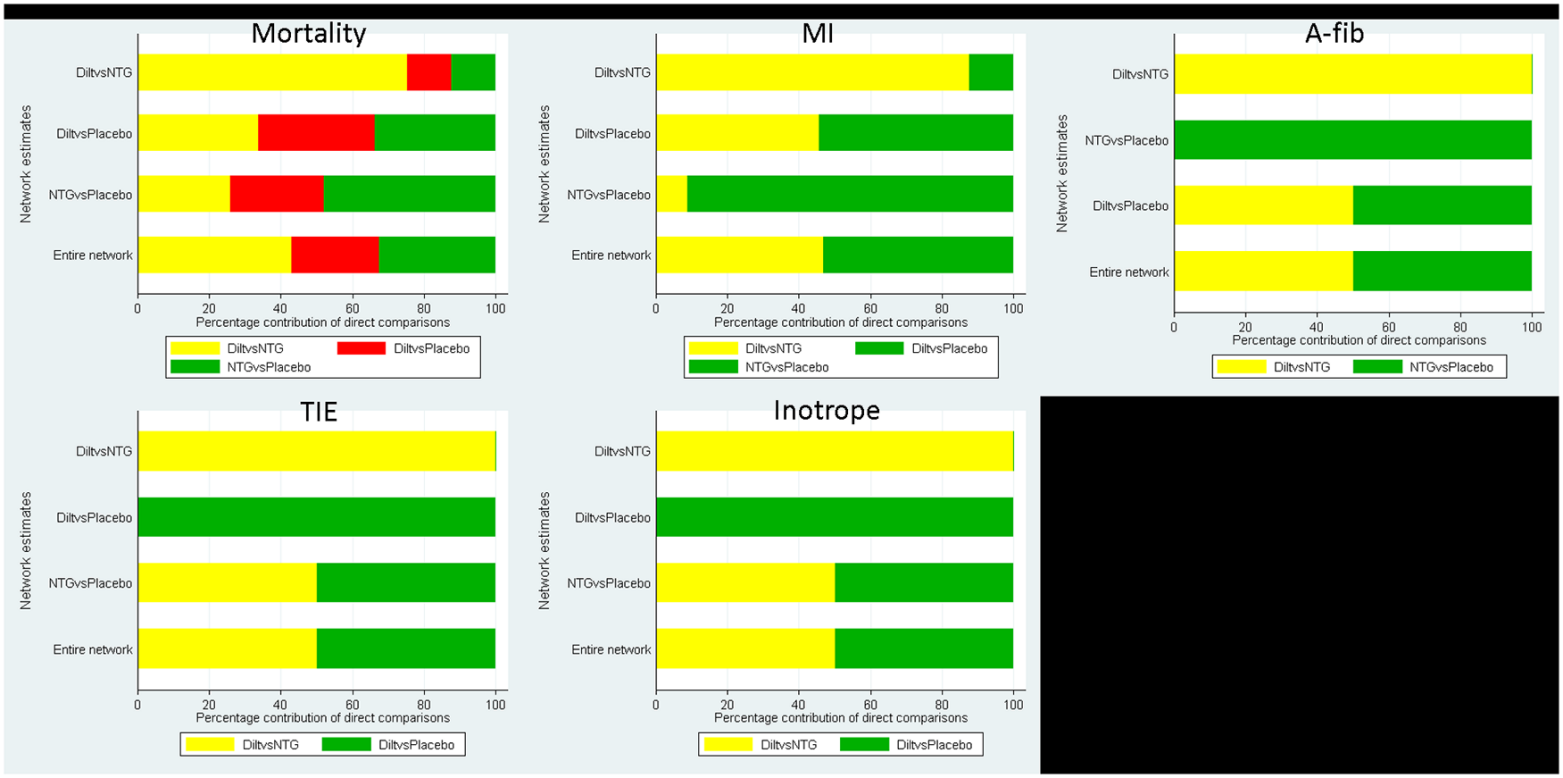
Network estimate for risk of bias for primary outcomes.

Two reviewers (XZ and XY) independently assessed concealment of allocation, blinding, and adequacy of analyses. Table 2 presented the quality assessment results using the Cochrane risk of bias assessment tool, with a score ranging from 5 to 7. Note that risk of bias can differ across different outcomes of interest, as each outcome draws from a different subset of studies. To ensure the relative contributions of different sources of direct evidence are accounted for appropriately, we presented risk of bias for each network estimate that integrated pairwise comparisons for primary outcomes. In Fig 3, the colors represent the risk of bias (green: low, yellow: moderate, red: high).

**Table 2:** Risk of bias for enrolled studies.

	Selection bias		Performance bias	Detection bias	Attrition bias	Reporting bias	Other bias
Study List	Random sequence generation	Allocation concealment	Blinding of participants and personnel	Blinding of outcome assessment	Incomplete outcome data	Selective reporting	Other sources of bias
Donegani 1986	high risk, prospective non-randomized	unclear	low risk	low risk	low risk	low risk	low risk
Hannes1993	low risk	unclear	low risk	low risk	low risk	low risk	low risk
Seitelberger 1994	low risk	unclear	low risk	low risk	low risk	low risk	low risk
Hannes1995	low risk	unclear	low risk	low risk	low risk	low risk	low risk
Keilich 1997	low risk, "patients were randomly assingned to …"	unclear, did not specify	low risk, the outcome is unlikely to be affected by not complete blinding	low risk, not blind record review, however unlikely to be influenced	low risk	low risk	low risk
Malhotra 1997	low risk "random assignement…done"	unclear, did not specify	low risk, the outcome is unlikely to be affected by not complete blinding	low risk, record review, unlikely to be influenced by not blinding	low risk	low risk	low risk
Lischk 1997	low risk	low risk	low risk, double blinded	low risk, double blinded	low risk	low risk	low risk
Hirnle 2000	low risk	unclear	low risk	low risk	low risk	low risk	low risk
Shapira 2000	low risk, last digit of medical record number	unclear	low risk	low risk	low risk, missing data in 16/161, however, long term outcomes not included in meta- analysis	low risk	low risk
Lassnigg 2001	low risk randomly assigned	unclear	low risk	low risk	unclear, one patient excluded after procedure	low risk	low risk
Zhang 2003	unclear, randomization done with date of surgery	unclear	low risk	low risk	low risk	low risk	low risk
Tabel 2003	low risk	unclear	low risk	low risk	low risk	low risk	low risk
Colso 1992	low risk	low risk	low risk, double blinded	low risk	low risk	low risk	low risk
Zanardo 1993	low risk	unclear	low risk	low risk	low risk	low risk	low risk
Armano 1995	low risk	low risk	unclear	unclear	low risk	low risk	low risk
Babin-Ebell 1996	low risk	unclear	low risk	low risk	low risk	low risk	low risk
Yavuz 2002	low risk	unclear	low risk	low risk	low risk	low risk	low risk
Fansa 2003	high risk, prospective non-randomized	unclear	low risk	low risk	low risk	low risk	low risk
Erdem 2015	low risk	unclear	low risk	low risk	low risk	low risk	low risk
Thomson 1984	low risk	unclear	low risk	low risk	low risk	low risk	low risk
Gallag 1986	low risk	low risk	low risk, double blinded	low risk	low risk	low risk	low risk
Withington 1988	low risk	unclear	low risk	low risk	low risk	low risk	low risk
Lell 1993	low risk	unclear	low risk	low risk	low risk	low risk	low risk
Knothe 1993	low risk	low risk	low risk	low risk	low risk	low risk	low risk
Apostolidou 1999	low risk, computer randomization	low risk	low risk	low risk	low risk	low risk	low risk
Chen 2000	unclear	unclear	low risk	low risk	low risk	low risk	low risk
Zvara 2000	low risk	low risk	low risk, double blinded	low risk	low risk	low risk	low risk

**Fig 4:**
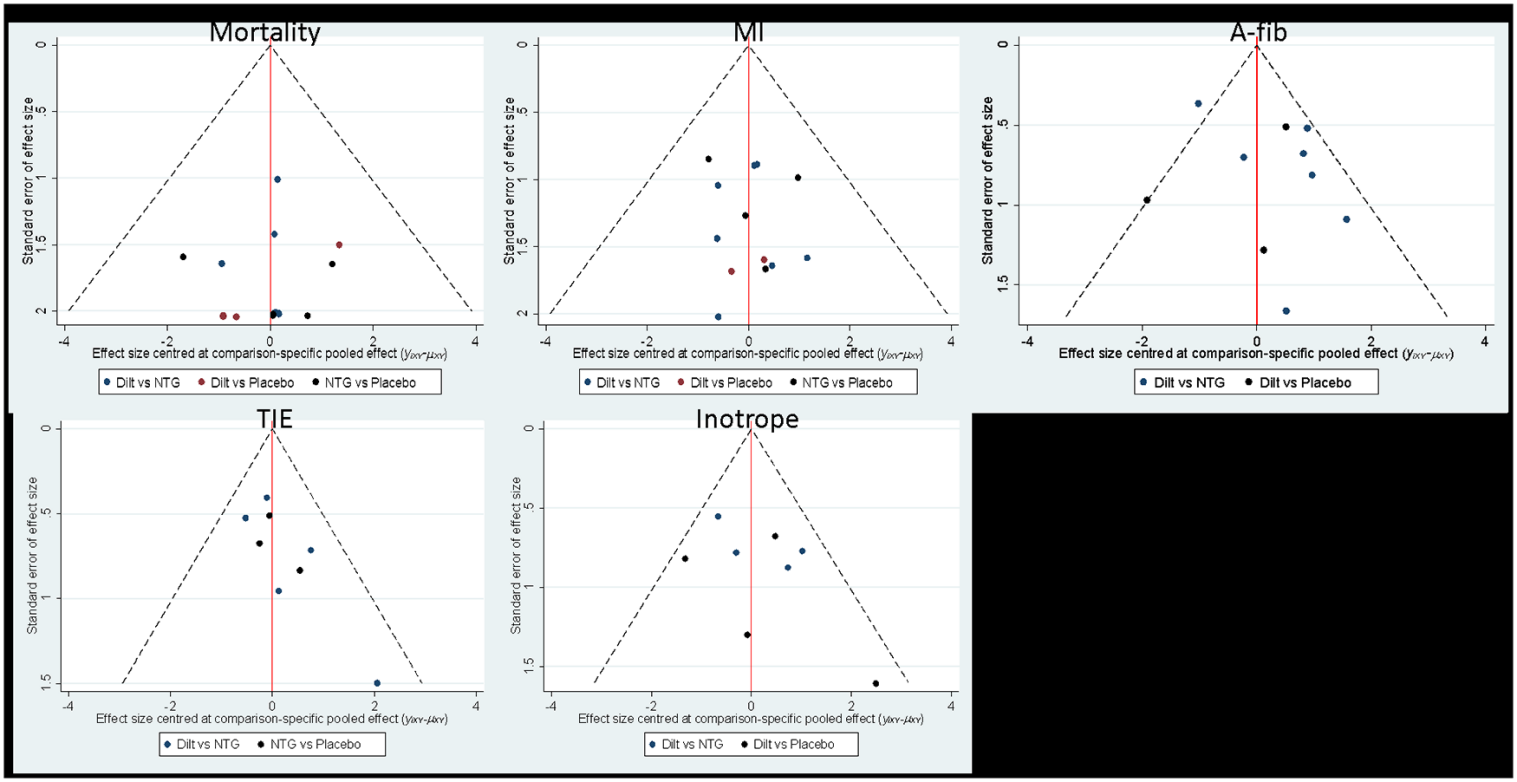
Funnel plots for primary outcomes.

For primary outcomes of closed-loop or open-loop network estimates, we presented funnel plots comparing any active intervention with non-intervention from pairwise studies. In Fig 4, we observed there were no indications of asymmetry on funnel plots of the pooled estimates, where different colors represent different pairwise comparisons.

### Perioperative mortality

**Fig 5:**
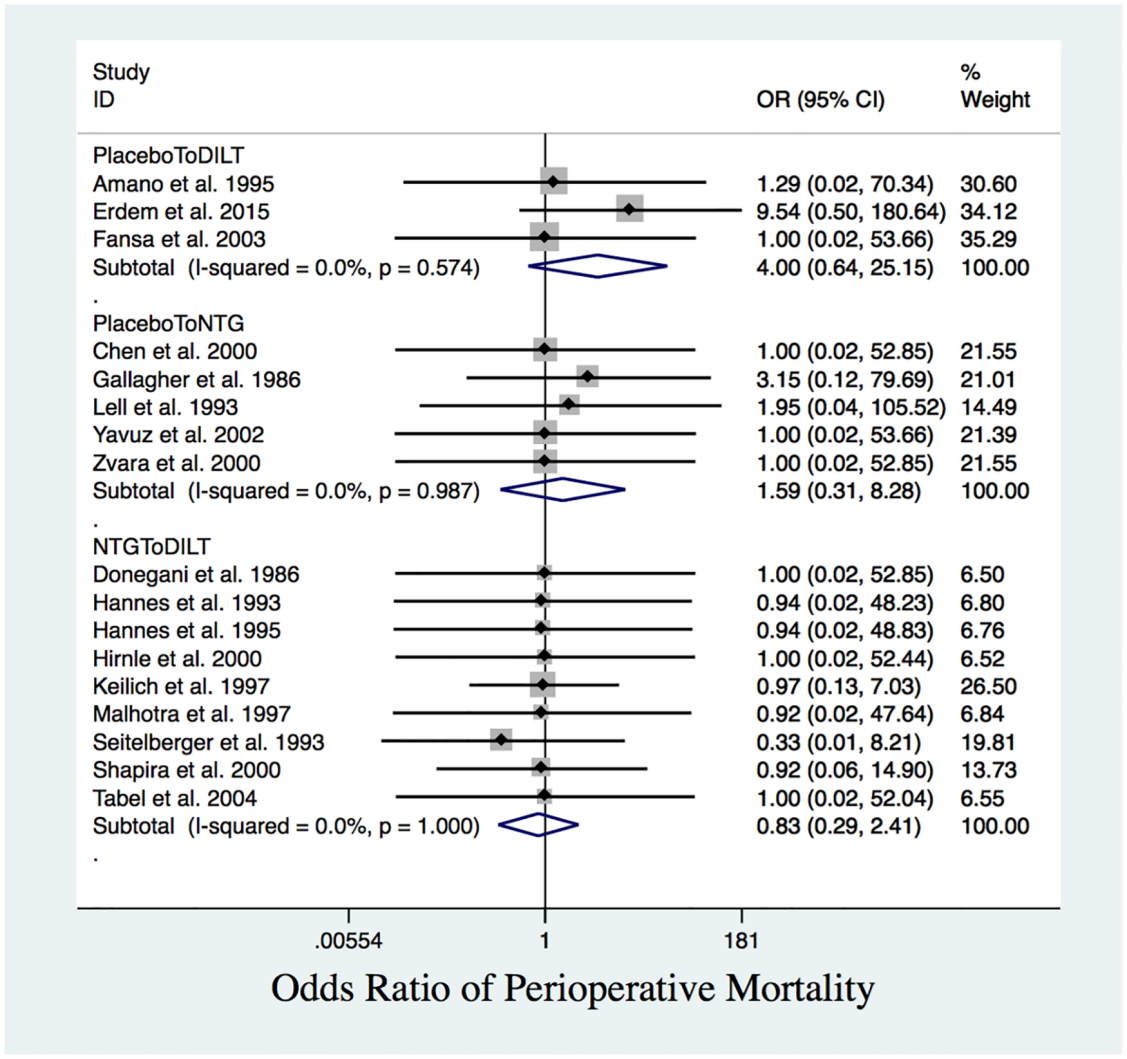
Perioperative mortality. Forest plot of OR of perioperative mortality. The differences among the interventions are statistically insignificant.

Pairwise comparisons did not reveal significant differences in perioperative mortality between patients receiving DILT, NTG or placebo (Table 3 and Fig 5).

**Table 3:** Pairwise comparison of perioperative outcomes.

**Pairwise meta-analysis (mortality)**				
**Included Studies**	Comparisons	OR (95% CI)	*I*^*2*^	P
**7 studies**	NTG vs. DILT	0.83 (0.29, 2.41)	0.00%	1.000
**5 studies**	Placebo vs. NTG	1.59 (0.31, 8.28)	0.00%	0.987
**3 studies**	Placebo vs. DILT	4.00 (0.64, 25.15)	0.00%	0.574
**Pairwise meta-analysis (Cardiac protection)**				
**Included Studies**	Comparisons	OR (95% CI)	*I*^*2*^	P
**post-MI**				
**7 studies**	NTG vs. DILT	1.83 (0.77, 4.30)	0.00%	0.975
**4 studies**	Placebo vs. NTG	2.24 (0.80, 6.28)	0.00%	0.596
**1 studies**	Placebo vs. DILT	6.20 (0.27, 141.32)	-	-
**TIE**				
**5 studies**	NTG vs. DILT	1.67(0.99, 2.82)	3.9%	0.384
**4 studies**	Placebo vs. NTG	1.42 (0.73, 2.75)	0.0%	0.707
**A-fib**				
**7 studies**	NTG vs. DILT	2.67 (1.15, 6.24)	62.20%	0.014
**2 studies**	Placebo vs. DILT	3.00 (1.18, 7.63)	0.0%	0.782
**Pairwise meta-analysis (Cardiac protection)**				
**Included Studies**	Comparisons	MD (95% CI)	*I*^*2*^	P
**CK**				
**1 study**	NTG vs. Placebo	-36.00 (-232.18, 160.18)	NA	NA
**5 studies**	DILT vs. NTG	-90.29 (-156.79, -23.79)	0.00%	0.691
**CKMB**				
**6 studies**	DILT vs. NTG	-12.47 (-18.33, -6.61)	63.5%	0.018
**1 study**	DILT vs. Placebo	-1.30 (-7.29,4.69)	NA	NA
**Trop-T**				
**4 Studies**	DILT vs. NTG	-0.66 (-0.87, -0.44)	0.0%	0.42
**Pairwise meta-analysis (Cardiac function)**				
**Included Studies**	Comparisons	OR (95% CI)	*I*^*2*^	P
**Inotrope**				
**4 studies**	NTG vs. DILT	1.78 (0.78, 4.07)	26.1%	0.255
**2 studies**	Placebo vs. DILT	0.19 (0.04, 0.73)	0.0%	0.419
**Pairwise meta-analysis (Cardiac function)**				
**Included Studies**	Comparisons	MD (95% CI)	*I*^*2*^	P
**CCI**				
**5 studies**	DILT vs. NTG	-0.02 (-0.18, 0.13)	21.3%	0.279
**2 studies**	NTG vs. Placebo	0.16 (-0.98, 0.42)	69.2%	0.072
**1 study**	DILT vs. Placebo	-0.10 (-0.62, 0.42)	NA	NA
**HR**				
**5 studies**	NTG vs. Placebo	2.54 (-6.22, 11.29)	95.1%	0.00
**1 study**	DILT vs. NTG	-13.00 (-23.56, -2.45)	NA	NA
**1 study**	DILT vs. Placebo	-9.40 (-18.88, 0.08)	NA	NA
**mBP (mmHg)**				
**3 studies**	NTG vs. Placebo	-2.61 (-11.70, 6.48)	98.30%	0.00
**1 study**	DILT vs. NTG	2.00 (-7.92, 11.92)	NA	NA
**1 study**	DILT vs. Placebo	1.90 (-8.54, 12.34)	NA	NA
**mPAP (mmHg)**				
**2 studies**	NTG vs. Placebo	-2.29 (-4.81, 0.23)	29.40%	0.23
**1 study**	DILT vs. NTG	-1.00 (-3.04, 1.04)	NA	NA
**PAWP (mmHg)**				
**4 studies**	NTG vs. Placebo	-1.05 (-1.43, -0.70)	0.0%	0.64
**1 study**	DILT vs. NTG	-0.50 (-2.97, 1.97)	NA	NA
**1 study**	DILT vs. Placebo	0.80 (-1.84, 3.44)	NA	NA

Note: OR greater than 1 favor the first treatment.

The network meta-analysis for mortality between two treatment groups and one placebo group (Table 4) implied that DILT, NTG and placebo were comparable when integrating the direct and indirect comparison results, where no inconsistency was detected (global test for inconsistency indicated *p* = 0.39). However, the SUCRA values implied that NTG and DILT were comparable, but better than placebo in preventing perioperative mortality (Table 5).

**Table 4:** Network meta-analysis results for mortality, post MI, TIE, A-fib, and inotrope.

Network meta-analysis (mortality), no inconsistency (p** = **0.39)**			
**OR (95%CI)**	**Placebo**	**DILT**	**NTG**
**Placebo (vs.)**	-	1.42 (0.39, 5.22)	1.37 (0.40, 4.64)
**DILT (vs.)**	0.70 (0.19, 2.57)	-	0.96 (0.35, 2.64)
**NTG (vs.)**	0.73 (0.22, 2.47)	1.04 (0.38, 2.81)	-
Network meta-analysis (post-MI), no inconsistency (p** = **0.99)**			
**OR (95%CI)**	**Placebo**	**DILT**	**NTG**
**Placebo (vs.)**	-	4.51 (1.34, 15.25)	2.26 (0.85, 5.99)
**DILT (vs.)**	0.22 (0.07, 0.75)	-	0.50 (0.20, 1.24)
**NTG (vs.)**	0.44 (0.17, 1.18)	2.00 (0.80, 4.96)	-
Network meta-analysis (TIE), no inconsistency (p** = **0.93)**			
**OR (95%CI)**	**Placebo**	**DILT**	**NTG**
**Placebo (vs.)**	-	2.21 (0.94, 5.21) *	1.43 (0.73, 2.77)
**DILT (vs.)**	0.45 (0.19, 1.07) *	-	0.64 (0.38, 1.11)
**NTG (vs.)**	0.70 (0.36, 1.36)	1.55 (0.90, 2.66)	-
Network meta-analysis (A-fib), no inconsistency (p** = **0.46)**			
**OR (95%CI)**	**Placebo**	**DILT**	**NTG**
**Placebo (vs.)**	-	2.86 (0.65, 12.61)	1.10 (0.20, 5.97) *
**DILT (vs.)**	0.35 (0.08, 1.55)	-	0.39 (0.18, 0.85)
**NTG (vs.)**	0.91 (0.17, 4.88) *	2.58 (1.18, 5.67)	-
Network meta-analysis (Inotrope), no inconsistency (p** = **0.14)**			
**OR (95%CI)**	**Placebo**	**DILT**	**NTG**
**Placebo (vs.)**	-	0.51 (0.16, 1.61)	0.28 (0.06, 1.21) *
**DILT (vs.)**	1.95 (0.62, 6.11)	-	0.55 (0.21, 1.41)
**NTG (vs.)**	3.57 (0.82, 15.51) *	1.83 (0.71, 4.72)	-

vs.: row versus column. OR less than 1 favor the treatment specified in the row; OR greater than 1 favor the treatment specified in the column;

*: indirect comparison;

**: p-value from global test for inconsistency.

**Table 5:** SUCRA scores for network meta-analysis.

SUCRA score (%)	DILT	NTG	Placebo
**Mortality**	65.0	68.6	16.4
**Post-MI**	94.4	52.8	2.8
**TIE**	94.8	46.1	9.1
**A-fib**	95.2	27.5	27.2
**Inotrope**	45.5	5.8	98.6
**CCI**	51.3	27.3	71.4
**HR**	97.0	11.8	41.2
**mPAP**	76.9	54.5	18.5
**PAWP**	46.4	87.3	16.3
**CK**	93.4	31.8	24.7
**CKMB**	77.9	3.5	68.6
**Trop-T**	100.0	0.0	NA
**mBP**	35.6	75.0	39.4

Note: the scores are inversely related to the frequencies of complications or the values of continuous variables.

### Cardiac protection outcomes

Four trials reported arterial graft flow measurements, but the data were insufficient for synthesis. One study revealed that, compared to placebo, continuous perioperative IV infusion of DILT significantly increased IMA blood flow[41]. Results from two other studies showed that patients receiving NTG had significantly higher blood flow in IMA or radial grafts[50,51], while a study comparing DILT to NTG showed that patients in the DILT group had significantly higher IMA blood flow[34].

**Fig 6:**
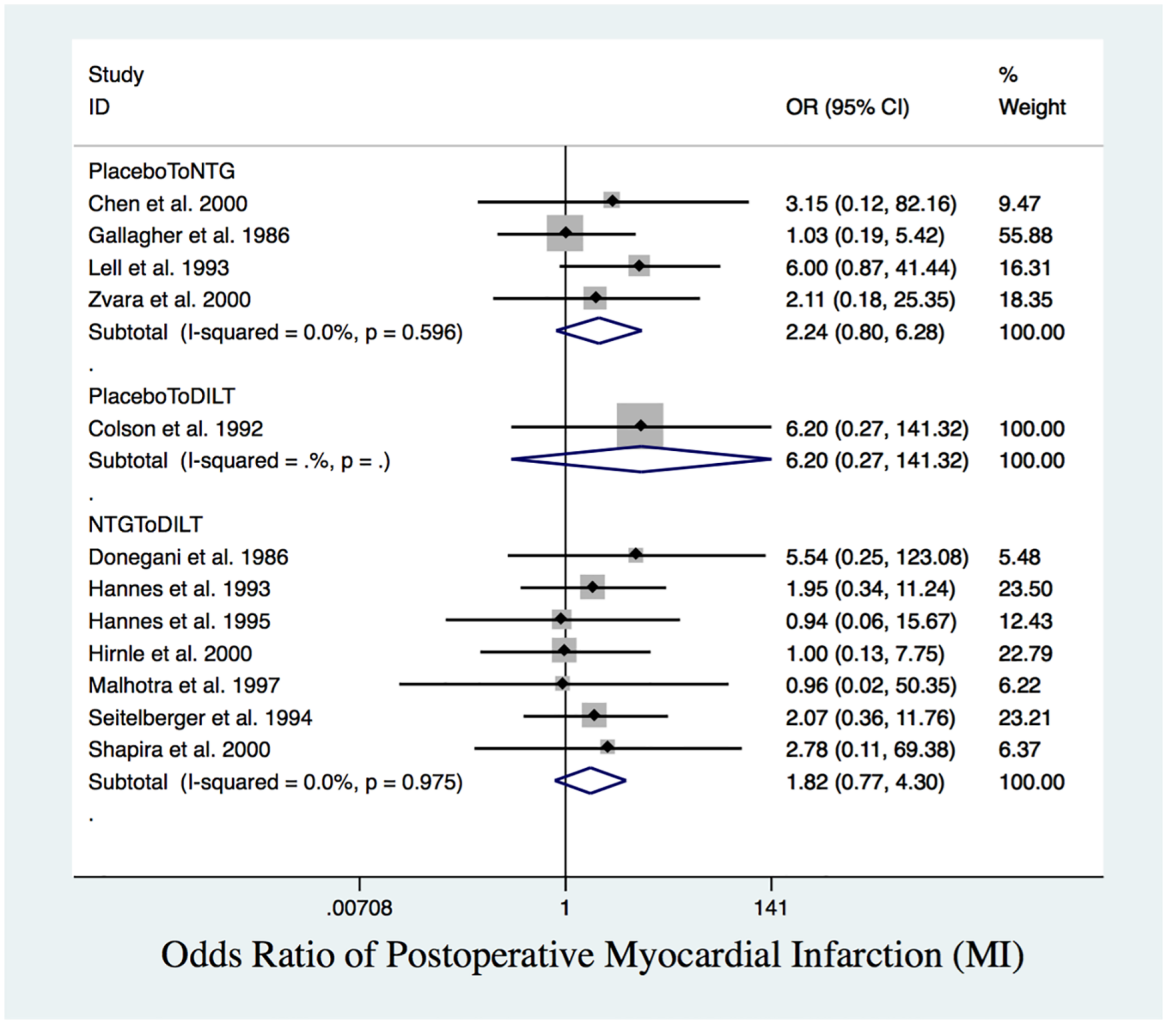
Postoperative MI. Forest plot of OR of postoperative MI. There was no significant difference in pairwise comparison between placebo and NTG, placebo and DILT, NTG and DILT.

From the NMA, post-MI had a closed triangle loop with no inconsistency (global test for inconsistency indicated *p* = 0.99). Although pairwise meta-analysis results for post-MI were not significant (Fig 6), network meta-analysis results revealed that, compared to DILT, placebo had higher rates of post-MI (OR = 4.51, 95% CI: 1.34 to 15.25) (Table 4).

**Fig 7:**
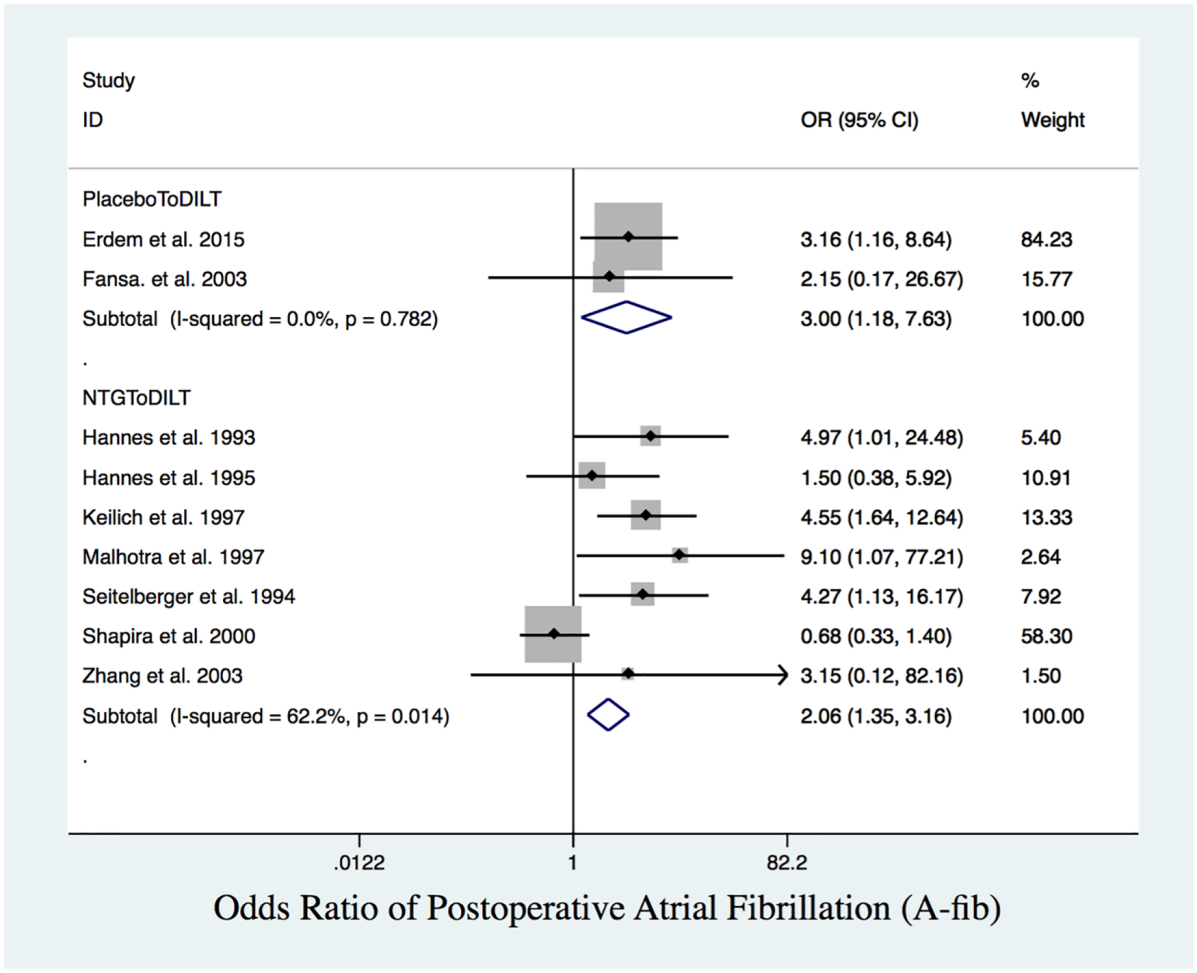
Postoperative A-fib. Forest plot of OR of postoperative A-fib. DILT had significantly lower odds than NTG and placebo.

Pairwise meta-analysis results showed that patients who received NTG (OR = 2.67, 95% CI: 1.15 to 6.24) and placebo (OR = 3.00, 95% CI: 1.18 to 7.63) had higher rates of postoperative A-fib than those who received DILT (Table 2 and Fig 7).

**Fig 8:**
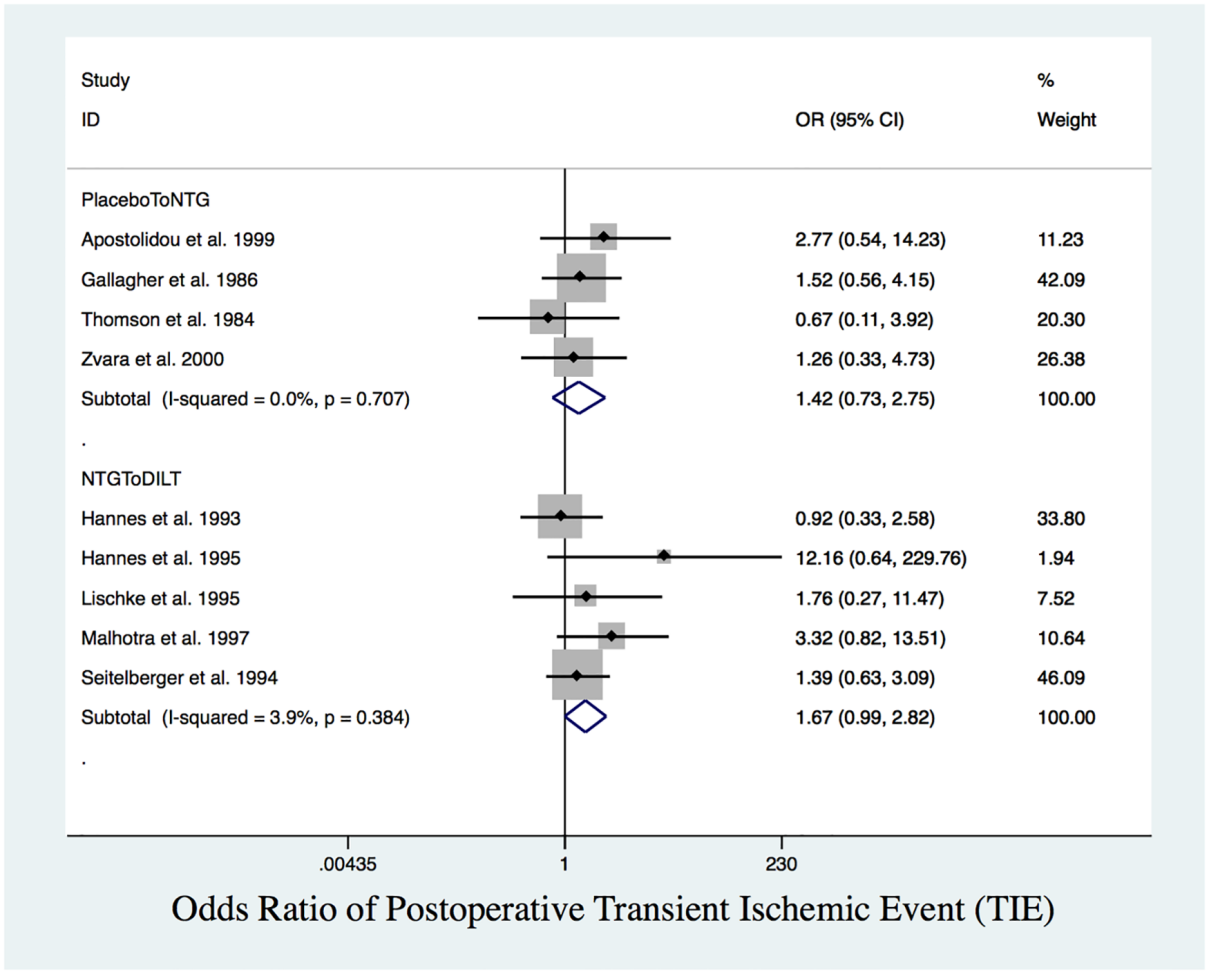
Postoperative TIE. Forest plot of OR of postoperative TIE. There was no statistically significant difference between placebo and NTG, NTG and DILT.

Since post-MI had a closed triangle loop with no inconsistency, we presented the network meta-analysis results by integrating direct and indirect evidence. For open triangle sloops (TIE, A-fib) with no inconsistency (global test for inconsistency indicated *p* = 0.93, *p* = 0.46, respectively), we presented both the direct estimates from conventional meta-analyses and the indirect estimates from network meta-analyses (Tables 4 and 5). From the NMA, TIE and A-fib had an open triangle loop with no inconsistency. Based exclusively on indirect comparisons (Table 4), placebo had a higher but statistically insignificant rate of TIE than DILT (Fig 8), while NTG had a significantly higher rate of A-fib (Fig 7) than DILT (OR = 2.58, 95% CI: 1.18 to 5.67).

The SUCRA values indicated that DILT ranked the highest in terms of protecting the heart from post-MI, TIE and A-fib (94.4%, 94.8%, 95.2%, respectively) (Table 5). The network meta-analysis results were consistent with the pairwise comparisons. The ranking from highest to lowest odds of post-MI, TIE and A-fib is placebo>NTG>DILT.

In addition, we observed that patients who received NTG also had higher post-operative peak cardiac enzymes: CK (MD = 90.29, 95% CI: 23.79 to 156.79), CKMB (MD = 12.47, 95% CI: 6.61 to 18.33) and Troponin (MD = 0.66, 95% CI: 0.44 to 0.87) than DILT (Table 2).

### Cardiac function outcomes

**Fig 9:**
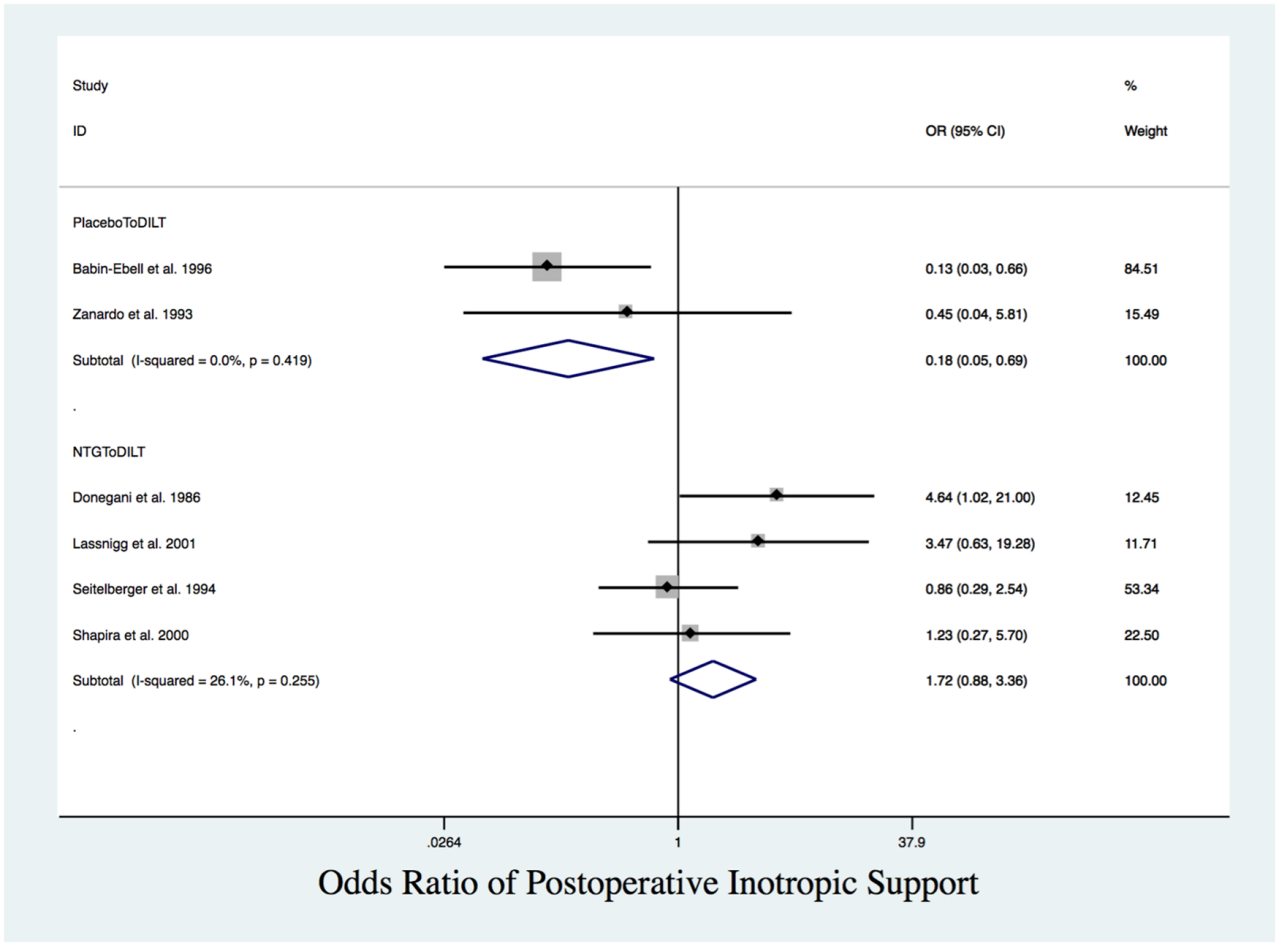
Requirement of postoperative inotropic support. Forest plot of OR of postoperative inotropic support between placebo and DILT, NTG and DILT. Patients who received DILT had significantly higher odds of needing inotropic support compared to those who received placebo.

In pairwise meta-analyses, patients treated with DILT had significantly lower post-operative HR than those with NTG (MD = 13, 95% CI: -23.56 to -2.45). Patients who received placebo had lower PAWP compared with NTG (MD = -1.05, 95% CI: -1.43 to -0.70). Compared with DILT, patients who received placebo had lower odds of requiring postoperative inotrope support (OR = 0.19, 95% CI: 0.04 to 0.73). Among the interventions, there was no significant difference in CCI or mBP (Table 2 and Fig 9). Based exclusively on indirect comparisons with no inconsistency, we observed that NTG patients needed more inotropic support compared to placebo as well, although the result was not significant. The SUCRA score ranking from highest to lowest indicated rates of needing inotropic support is NTG> DILT>placebo (Table 5).

## Discussion

To summarize the results of our current conventional and network meta-analyses, intraoperative measurements indicated that both DILT and NTG increased graft blood flow, which may help preventing graft spasm. However, the data are insufficient for synthesis. Compared with placebo, patients who received perioperative continuous IV infusion of DILT had significantly lower odds of postoperative cardiac ischemia and A-fib, but patients on DILT may need more inotropic support compared to placebo. Compared with NTG, the significantly lower HR and postoperative peak cardiac enzymes in patients on DILT indicates that DILT may be superior to NTG in preventing postoperative cardiac ischemic injuries.

The goal of CABG is to restore blood supply to ischemic heart through arterial and venous grafts. Graft spasm during and early after the procedure has negative impacts on the cardiac function and hemodynamic stability. CCBs and nitrates are two major categories of coronary vasodilators that were investigated for preventing graft spasm. Among the CCBs, DILT, a benzothiazepine, and verapamil, a phenylalkylamine, were considered suitable for perioperative continuous IV infusion due to their negative chronotropic effect. Studies *in vitro* and *in vivo* have consistently demonstrated that CCBs, topically applied or directly injected into the grafts, can significantly increase blood flow in human IMA and the radial artery (RA) grafts. Data from studies by Erdem[41] and Tabel[34] showed that continuous IV infusion of DILT is superior to both placebo and NTG in improving blood flow in dissected left IMA and RA segments before graft anastomosis. Although there were no data of direct flow measurement postoperatively, the lower incidence of cardiac ischemia and cardiac arrhythmia from the current meta-analysis indicated that coronary blood flow was likely better maintained early postoperatively in patients who received perioperative DILT infusion.

Our meta-analysis showed that intraoperative infusion of DILT resulted in significantly lower odds of postoperative A-fib. This is particularly interesting as the most recent ACCF/AHA guideline for CABG in 2011 states that nondihydropyridine CCBs such as DILT can be useful to control the ventricular rate in the setting of A-fib but are not indicated for prophylaxis[52]. However, this recommendation was based on one meta-analysis published in 1991[53]. In that study, verapamil, a nondihydropyridine CCB, failed to show a protective effect against the development of supraventricular arrhythmias (SVAs) after CABG. The discrepancy between that meta-analysis and ours may be due to the differential efficacy of oral verapamil and intravenous DILT against SVAs and the significant improvements to surgical and perioperative managements over the years. The results from our meta-analysis suggest that DILT might be useful for prophylaxis against A-fib after CABG.

The mechanism of cardiac protection by DILT is likely multifactorial. DILT inactivates cell surface L-type calcium channels, preventing the extracellular influx and sarcoplasmic release of calcium ions, promoting smooth muscle relaxation and therefore dilation of native and grafted coronary vessels[54]. Additionally, experimental data suggest that DILT could be involved in regulating endothelial function. DILT reduces the blood concentration of endothelin-1, a potent vasoconstrictor released from vascular endothelium, which may promote vascular smooth muscle relaxation through nitric oxide (NO) related signaling pathways[55,56]. In fact, there is evidence suggesting DILT could up-regulate NO synthase gene expression in endothelial cells[57]. Besides increasing blood supply through dilating coronary vessels, DILT may also have significant anti-inflammatory effects by regulating pro- and anti-inflammatory cytokines[40,58]. Clinically, Haak et al.[59] found that endothelin-1 level in circulation was elevated during and immediately after CABG, and perioperative DILT IV infusion significantly reduced endothelin-1 release. Compared to those who received NTG, patients who received DILT had more favorable hemodynamic status early postoperatively.

Nitrate family molecules, including NTG, have been applied clinically to relieve acute and chronic angina pectoris since 1876. Its mechanism was found to be NO mediated vasodilation of coronary arteries to improve oxygen supply to ischemic cardiac muscle, and afterload reduction to decrease oxygen demanding from the heart[60–62]. Previous studies confirmed that topical application and intravascular injection of NTG had potent vasodilatory properties[63]. However, despite their effectiveness in relieving acute chest pain, the long-term outcome benefit of nitrates in patients with CAD remains questionable. It is well documented that patients continuously taking nitrates quickly develop significant tolerance to the drugs, which not only diminish the treatment effects, but also had the tendency of causing more cardiac ischemic events[64]. Nakamura et al.[65], found that chronic usage of nitrates in patients with CAD may be associated with increased mortality. A large-scale retrospective study showed that pre-operative IV infusion of NTG failed to provide short term outcome benefits in patients who underwent CABG for unstable angina, patients on preoperative NTG also required prolonged postoperative mechanical ventilation and had more acute cardiovascular events[66]. The results from the current meta-analysis also suggest that perioperative continuous IV infusion of NTG had less cardiac protective effects in patients undergoing CABG compared to DILT; patients receiving NTG are more likely to have cardiac arrhythmic events compared to placebo and DILT. Therefore, perioperative continuous IV infusion of NTG may not be beneficial for patients having CABG.

In this study, network meta-analysis was used to compare treatment effectiveness of three treatment groups. The results suggest that DILT has the best protective effects against cardiac ischemia and arrhythmia in patients undergoing on-pump CABG. The advantage of network meta-analysis, an extension of traditional pairwise meta-analysis, is that it has the advantage of comparing multiple treatments with few or no head-to-head comparison data available. It can also help to determine the best available treatment and provide clinical guidelines. By “recycling” the data from prior studies, network meta-analysis is a cost-efficient statistical tool for comparing multiple interventions.

The current systematic review and network meta-analysis has some limitations, however: Firstly, due to the heterogeneity in the designs of the original studies, many critical outcomes cannot be evaluated because of unavailable or insufficient published data. Secondly, the sample sizes in most of the enrolled studies are relatively small, as we did not enroll unpublished data. This limitation in the quantity and quality of data could affect the power of the pairwise and network meta-analyses. Thirdly, the studied population in the enrolled studies were elective patients whose clinical conditions were relatively stable. Clinically more complicated, unstable patients were mostly excluded before or during the studies. This may be a confounding factor for the analyses of perioperative mortality, as it suggests that the conclusions drawn from the current meta-analysis may not apply to patients with different severities of clinical conditions. Most importantly, the studies enrolled were from the last three decades, an extensive period that has seen significant improvements in surgical techniques, perioperative and long-term medical managements of the patients undergoing on- or off-pump CABG. Studies have shown significant reduction of mortality as well as improvement of short- and long-term outcomes[67,68]. Due to the unavailability of anatomical evidence supporting the relief of graft spasm after closing of sternum as well as insufficient long-term follow-up data of DILT and NTG applied perioperatively, the overall outcome benefits of perioperative continuous IV infusion of DILT and/or NTG in patients undergoing on-pump CABG remains uncertain.

In conclusion, the current systematic review and network meta-analysis suggests that, compared to NTG and placebo, perioperative continuous IV infusion of DILT had stronger protective effects against postoperative ischemic cardiac injuries and A-fib. Possibly, DILT’s outcome benefits may be due to increased blood flow in arterial grafts. However, compared to placebo, patients may need more inotropic support.

## Supporting information

S1 Table Ovid MEDLINE search from 1946 until Feb 2018 without language restriction. Comprehensive database search, including the components of “Epub Ahead of Print” and “In-Process & Other Non-Indexed Citations”, was conducted initially on 11/15/2016, the search had been continuously updated monthly until the date when the manuscript was submitted.
(DOCX)

S2 Table PRISMA 2009 checklist. The complete 27 checklist items pertain to the content of the current systematic review and network meta-analysis. Items s# 16, 23 and 27 are unfilled because there are no additional analyses conducted, there is no institutional funding support for this study.
(DOC)
