# Supplementary material for: Perioperative diltiazem or nitroglycerin in on-pump coronary artery bypass: A systematic review and network meta-analysis
Source: PLoS One. 2018 Aug 30;13(8):e0203315. doi: 10.1371/journal.pone.0203315 (PMC6117025; doi:10.1371/journal.pone.0203315)
Supplement: S1 Table — Comprehensive database search, including the components of “Epub Ahead of Print” and “In-Process & Other Non-Indexed Citations”, was conducted initially on 11/15/2016, the search had been continuously updated monthly until the date when the manuscript was submitted. (DOCX) [file pone.0203315.s001.docx]

| Line number | Search terms include keywords and standard MESH heading/subheadings |
| --- | --- |
| 1 | “cardiac surgery” OR “Thoracic Surgery” |
| 2 | “coronary artery bypass” OR “Coronary Artery Bypass” |
| 3 | “cardiopulmonary bypass” OR “Cardiopulmonary Bypass” |
| 4 | “cardiac surgical procedure” OR “Cardiac Surgical Procedures” |
| 5 | “calcium channel blocker” OR “Calcium Channel Blockers” |
| 6 | “diltiazem” OR “DILTIAZEM” |
| 7 | 5 OR 6 |
| 8 | “nitrate” OR “NITRATES” |
| 9 | “nitroglycerin” OR “NITROGLYCERIN” |
| 10 | 8 OR 9 |
| 11 | 1 OR 2 OR 4 |
| 12 | 3 AND 11 |
| 13 | 7 AND 12 |
| 14 | 10 AND 12 |
| 15 | 13 OR 14 |
